# Revisiting fertility regulation and family ties in Tunisia

**DOI:** 10.1186/s12884-023-05408-9

**Published:** 2023-02-01

**Authors:** Olfa Frini, Christophe Muller

**Affiliations:** 1 Institut Supérieur de Comptabilité & d’Administration Des Entreprises, Manouba University, Tunis, Tunisia; 2 Aix-Marseille Univ, CNRS, AMSE, Marseille, France

**Keywords:** Fertility regulation, Age at marriage, Birth control, Family influence, Contraception, Tunisia

## Abstract

**Background:**

We revisit fertility regulation in Tunisia by examining the role of the extended family. As marriage is the exclusive acknowledged childbearing context, we examine fertility analysis in Tunisia through the sequence: woman’s marriage age, post-marriage delay in the first use of contraception, and past and current contraceptive use. We trace the family socio-economic influences that operate through these decisions.

**Methods:**

Using data from the 2001 PAP-FAM Tunisian survey, we estimate the duration and probability models of these birth control decisions.

**Results:**

In Tunisia, family ties and socio-cultural environment appear to hamper fertility regulation that operates through the above decisions. This is notably the case for couples whose marriages are arranged by the extended family or who benefit from financial support from both parental families.

**Conclusion:**

This calls for family planning policies that address more the extended families.

**Supplementary Information:**

The online version contains supplementary material available at 10.1186/s12884-023-05408-9.

## Introduction

Historically, the reduction in fertility rates has been a prerequisite for development and growth in almost all countries. In fertility studies the factors explaining the number of children through birth control decisions have received some attention, following a long tradition initiated by Bongaarts [1]. However, fertility decline, in general, occurs through reduced demand for children and use of birth control. This in turn, depends on couples’ positive attitudes and willingness to adopt contraception and on the availability and accessibility of fertility regulation techniques. To achieve their desired number of children, women deliberately alter their reproductive process using family planning. Therefore, factors affecting fertility can be investigated by studying factors affecting birth control.

Easterlin [2, 3] framework of fertility regulation determination involves three types of consideration: motivation, attitudes and access toward regulation. A couple’s “motivation” to control fertility is viewed as a function of the gap between actual and desired fertility. ‘’Gender’’ preferences, as studied in Karsten and Kohler [4] can also play a role in the use or not of birth control, for example so as to obtain at least a boy. Fertility becomes regulated when the disadvantages of unwanted children are greater than the cost of fertility regulation (including both subjective disadvantages and the economic cost). “Attitude” embraces both approval and disapproval of family planning. “Access” to birth control technique is perceived through the couple’s choice of contraceptive methods and on their availability and supplies. Based on these insights, a woman could adopt diverse birth control means over her reproductive span. We focus on three of these means, for which we have data.

First, exposure to unwanted birth may be controlled by delaying marriage. Marriage has long been considered to be a proxy for exposure to the risk of fertility, on the grounds that premarital sexual intercourse is relatively uncommon among women [5]. This is all the more relevant in Muslim countries, where marriage is the sole socially tolerated context for childbearing [6]. In particular, in Tunisia, the primary reason for getting married is to have children, with exceptional out-of-wedlock pregnancies. This is corroborated by access to birth control options, such as the pill, and abortion, reducing marriage incidence [7]. Besides, marriage timing may affect both the supply of and demand for children [8, 9]. Therefore, a woman who pursues personal projects, such as higher education and career achievement, has incentives to delay marriage.

A second regulation decision is the timing of the first contraceptive use after marriage. Contraceptive use may take place episodically along the reproductive span—for example, to allow for birth spacing that smoothes economic and time burdens across years. The first use may occur immediately after marriage if children are not immediately desired. The time gap between wedding and first birth control may entail both anticipated and unanticipated decisions, which reflect not only cost–benefit calculations but also subjective attitudes toward family planning. Nevertheless, this gap can easily be observed, as opposed to the detailed birth control process. This justifies investigating it in this paper.

A third, unobserved, stage of regulation is the full sequence of contraceptive uses by the spouses over the reproductive span. However, whether the woman is currently using contraception, or if she has used it in the past, can sometimes be observed. Of course, these decisions cannot occur before the first use of contraceptive techniques.

Often, it is assumed that the birth control is decided only by the woman; whereas, the husband or the extended families of the spouses may also be involved. In this paper, we track the encroachment of families in successive steps of birth control. To do this, we follow Bongaarts [10] and Bongaarts and Potter [11] who distinguish direct and indirect determinants of fertility. Direct determinants include exposure factors (such as being married), deliberate fertility control (e.g., contraception), and natural fertility factors. The indirect determinants refer to cultural, socio-economic and environmental factors. Among all these determinants, Bongaarts emphasizes four primary proximate factors that generate salient differences in fertility levels across societies. They are: marriage—notably the typical age at marriage -, contraceptive habits, lactation and induced abortion. We follow this approach while focusing on his first two factors. His last two factors (abortion and lactational infecundability) are omitted because they are not measured in our data. However, one major difference from Bongaarts is the systematic attention we devote to extended family role in these factors from a post-marriage perspective.

In the Tunisian case, we pursue the idea that fertility outcomes may result not only from wife and husband decisions, but also from the strategies of the extended family. We define the ‘extended family’ as composed of the nuclear family plus grandparents, granduncles and grandaunts, and other relatives potentially influential for fertility decisions. That is: dwelling in the same homestead is not imposed. The social control of elders on a young couple’s procreation may be strong in traditional families. This influence pattern may combine with the traditional authority of the father on his wife and his children. These cultural features express the long-standing moral dominance of the extended family on the individual in Tunisia.

However, the typical Tunisian family has gradually changed, and is still changing. The family has become nuclear in most cases, although the young married couple still usually lives in areas near their parents, which preserve their close relationship. In these conditions, conventional symbolic and ideological family values, and their counterpart that is potential assistance from elders, often remain relevant and extant. Even though procreation is less and less controlled by the extended family, the latter still exerts some leverage in this area, as we shall show. Ben Salem [12] claims that the control of the procreation by the extended family is still present in certain regions, in certain socio-professional activities, or elsewhere as a residual of an old state of affairs, like a surviving traditional feature. This is what we explore in this paper.

We show how some insight can be gained by considering the above successive birth control decisions made by women in Tunisia, or on their behalf by their husband or their families. Indeed, the likelihood of new births not only depends on the mother’s biological characteristics, her preferences and her economic activity, but may also be affected by her family context, including within the extended families of the spouses. These family networks may influence various facets of young couples’ lives, for example when choosing the spouse, financing the wedding, or determining the number and education of the offspring.

The PAP-FAM survey 2001 allows us to track family mutual support and intrusion. Although these data are not recent, they are still the only ones, in MENA or elsewhere, that provide unique information about the role of family networks in birth control. Therefore, these data are still of interest. Moreover, there are stable features in the fertility landscape of Tunisia: Muslim family rules, the almost exclusion of births outside marriage, and a persistent presence of the extended family around the procreation process, are among them. As a consequence, marital women are still the relevant population to consider to study birth control. As before, very few sexual relationships take place outside marriage, at least as far as they can be observed. Finally, the ISF (synthetic general fertility index) has been relatively stable around 2.1 children per women since the data year (2.1 in 2001, temporarily raising up to 2.4 in 2014, according to the national census, and dropping back to 2.1 in 2019, according to the MICS2019 survey). Besides, even though the nuptiality rate (number of marriages during the year divided by the population) has dropped recently to its lowest at 12.1 percent in 2021, it has been accompanied by a parallel drop in the use of contraceptive. Then, these changes seem to correspond to a relative stability of the contraception process of married women.

Nonetheless, childbearing has evolved over the last twenty years. For example, never-married women are more numerous, with a steady rise of the celibacy rate up to 60 percent for the 34–45 years old women in 2020. Abortion is also on the rise, whereas the contraceptive prevalence rate has diminished from 63 percent in 2001 down to 51 percent in 2018. In spite of these changes, to understand the future, it is important to understand where it does come from, and examining the available data remains worthwhile. In particular, the persistent presence and role of the extended family is not likely to vanish completely soon, partly because it increasingly benefits from new channels through internet social networks (e.g., Facebook), from the rise of radical Islamic ideologies, and from daughters remaining longer at the parents’ home.

In 2001, 90 percent of married couple keep up in touch with family (60 percent permanently and 30 percent occasionally), 26 percent cohabit with family, 48 percent benefit from familial financial support, 13 percent from familial wedlock fee, 20 percent from family childcare. Conversely, 71 percent of husbands (respectively, 48 percent permanently and 23 percent occasionally) support elderly members after their own family establishment (5 percent for wives). In addition, family is the original meeting place of the future spouses for 63.5 percent of couples, while 42.3 percent marry with first cousins or other kinship. Of course, all these dimensions are likely to be highly correlated. Thus, they can be considered as diverse proxies of the general extent of a family influence, which may affect birth control decisions as well. At least, this is our working hypothesis since the survey did not ask about family involvement in family planning.

All birth control decisions entail expectations, trials and errors. Moreover, the analysis of these decisions may be further complicated by changes in individual preferences, and shifting tradeoffs between regulation motives, along with each woman’s lifecycle. Specifically, schooling prospects, labor force participation, family founding, and old age health concerns may, in a somewhat successive fashion, occupy the minds of women as they age. These concerns may generate postponement of parenthood and fertility outcomes, as precisely studied by Nitsche and Brückner [13] for highly educated US women, and similar phenomena may take place in Tunisia. Faced with this complexity, it is clear that estimating a complete structural fertility model, at least in the Tunisian case, is far beyond what is possible with the available cross-sectional data. In these conditions, our approach is instead to focus on the observable birth control decisions and their suggestive relationships with observed covariates, notably with family variables, which is the main goal of the analysis.

The structure of the paper is as follows. Sect. "Fertility regulation in Tunisia" presents the context and the data. Section 3 reports and discusses the results. Finally, Sect. 4 concludes.

## Materials and methods

### Fertility regulation in Tunisia

Fertility has plummeted in Tunisia over the last half century. According to the Tunisian Annual Statistics of the National Institution of Statistics (NIS) [13], the fertility rate, which was close to eight children per woman in the early 1960s, was nearly below the renewal threshold (2.05 children per woman) in 1999. Although a slight rise has been recorded since 2010, the fertility rate remains low at 1.8 children per woman in 2021. These demographic changes have been fostered by laws and institutions that have enhanced the social and legal status of women, starting with the 1956 Code of Personal Status that promoted female emancipation [14]. The Code regulated marriage and divorce, abolished polygamy, set a minimal legal age for marriage, and replaced repudiation with divorce. The minimal legal age for marriage was set to 15 years for women and 18 years for men in 1956 and later further revised in 1964 to 17 and 20 years, respectively. As a consequence, women and men in 2014 married on average at 28 and 34 years of age, respectively. These changes have had direct consequences on fertility because out-of-wedlock births only amount to 0.5 percent of births over 2000–2012 [15] (referring to the annual report on children: 2000, 2012 of Tunisian Ministry of Women and Children).

From the independence in 1956, the Tunisian government encouraged families to limit their number of children through public campaigns. Advertisement and sale of contraceptive devices were legalized in 1961. A new institution, the ONFP (National board of Family and Population) was created in 1964, with its core mission to help the government limiting and monitoring fertility. Abortion was legalized in 1973without conditions to fulfill. Finally, modern contraceptive instruments allowing couples to better control their progeny were provided for free.

Family planning policies contributed to the rise in the contraceptive prevalence rate from 31 percent in 1978 to 62 percent in 2001, before it declined down to 51 percent in 2018. The 2001 ONFP survey report [16] claims that 82 percent of respondents stated that they had been using contraceptives at that time and before. No major differences occurred across regions (e.g., 75 percent for rural vs. 83 percent for urban areas), or across education levels (75.9 percent for illiterate, 81.6 percent for primary, 84.8 percent for secondary, and 83.4 percent for higher education). As a consequence, the number of surviving children at the first birth control use collapsed. The younger observed women, aged 20–24 years, often started to regulate their fertility after giving birth to a single surviving child. For them, the average delay before the first birth control steadily fell between 1978 and 2001, from 6.56 years to 1.37 years. On average, regulation takes place after about two (1.93) surviving children (1.61 in urban areas vs. 2.63 in rural areas). The most commonly used methods are IUD (intra-uterine device, 44.1 percent), the pill (17.4), tying tubes (16.7), and calendar (11.8). The contraceptive prevalence rate that was almost nil in the sixties, rose to above 30 percent in the seventies, to reach 59.7 in 1994 and 70.5 in 1999, then fell down to 63 in 2001, and 51 in 2018.

Gastineau and Sandron [17] discuss the Tunisian Family Planning policy over 1964–2000, and this discussion is completed in Gataa [18] up to 2014. Initially, the policy was focused on a contraception after the desired number of children has been obtained, early after marriage. From 1976, sophisticated developments occurred with enhanced pills, which never reached the popularity of the DIU. In the early eighties, the Sixth Development Plan strengthened family planning. Since most effects of delaying marriage had already been reached at this stage, all the efforts were devoted to the diffusion of contraception methods, including in the relatively neglected rural areas. From the mid-eighties, family health issues come to the ONFP policy forefront, diverting resources formerly devoted to contraception (Maffi and Affes [19]). From the late nineties, the effectiveness of the ONFP contraception services has declined. All along the long spell of contraception policies in Tunisia, the role of the extended family has been mostly overlooked.

Another factor of the relative decline in modern contraception is the progressive surge in abortion services that compete with it, as the second most important birth control method after DIU. This partly explains why young women have used modern contraception methods less than their elders. Gastineau [20] reviews the changes in birth control modes during the Tunisian demographic transition. She notes the increasing number of women using abortion, post 1973 legalization, even though they were generally aware if modern contraception. However, abortion is still a taboo subject among young women, which may explain why this information was not collected during the survey.

These policies and other development policies in Tunisia have greatly disrupted the influence of the extended family. Over time, protection and insurance family roles were much substituted with state institutions such as social security. In parallel, the incentives for child work, especially on farms, collapsed with the development of modern market activities and the increase in living standards. The separation of economic activities from the family led to a drop in cohabitation of the different generation, which boosted the independence of the married couple from their relatives. Growing living standards reduced the need for support from grandparents, and rising female worker participation on the labor market made the couples more self-sufficient. The ONFP promotion campaigns for birth control contributed to the decline of traditional values. This was the continuation of President Bourguiba’s strategy of transforming the country by morally modernizing its families. From the Code du Statut Personnel in 1956, the success of birth control and family planning strategies have always rested on a heavy public advertisement effort.

Moreover, the rise in contraceptive availability and use sustained the procreative autonomy of the nuclear family from the pressures of the extended family. Thus, the declining influence of the extended family in the procreation process was associated with the demographic transition in Tunisia. Once the straightjacket of the traditional family, as the organization controlling the family production and procreation processes, is relaxed, couple can feel free to reduce the size of their offspring, and to use birth control techniques to attain this objective. However, extended families are still around and potentially influential.

It has been suggested, e.g. in Rindfuss et al., [21] for Norway that parents may choose to dwell in locations where childcare opportunities are available and that these moves contribute to explain birth timing. This is consistent with Tunisia’s couples tending to live close to their parents’ residence location.

### The data

The data we use are taken from the 2001 PAP-FAM Tunisian survey. Its nationally representative sample was drawn into 360 clusters, each one of 20 households, with stratification by governorate and rural/urban areas. Beyond its focus on family relationships, social culture, and fertility behavior, the rich survey’s questionnaire provides information on various household characteristics, including reproductive and sexual health, for 6702 households. After dropping a few non-responses (response rate of 92 percent) and focusing on the population of interest, we obtain a sample of 3175 married women from 15 to 49 years old, a reasonable approximation of their fertile spell. The main population of interest when studying fertility regulation is that of married women. The women in the survey span several decades when they got married. Dividing the sample by age group would have been interesting, but this is not fructuous with the limited sample size and the number of independent variables that we want to include.

Data truncation problem arises in our married women sample since not all women marry. However, the proportion of never-married women is small (only 3.9 percent in 2001, at the time of the survey, 1.6 in 1984 and 2.3 in 1994 [22]), and we can neglect this source of bias, which we cannot correct anyway. Moreover, in 2001 (respectively, 1984), only 9.1 (1.6) percent of the 45–49 aged women were not married, and 6.8 (1.5) percent for the 50–54 aged women [23]. Another issue, specific to the marriage’s age equation, is that some women are not yet married, and therefore not surveyed in the PAP-FAM survey. We deal with this truncation issue below, which turns out to have little influence on the results in this case. Finally, access to public versus private family planning facilities is not distinguished, although the latter may have higher quality as found for example in Ethiopia by Tessema et al. [24]. Let us now discuss the variables used in the analysis.

As aforementioned, the dependent variables describe the birth control decisions that we can observe in our data: the woman’s marriage age, the post-marriage delay in the first use of contraception, and the contraceptive use. Specifically, two dependent variables depict respectively a *woman’s age at marriage* and her *marriage duration before first birth control,* both in years*.* Moreover, two dependent dummy variables inform about the prevalence of birth control: the *contraceptive ever used* and the *contraceptive currently used*. The respondents state respectively whether they have used contraception in the past and whether they are using contraception at the time of the interview.

The independent variables are inspired by the literature on the determinants of fertility, given the information available in the survey, and we complete them with original variables on family interactions. Finally, we maintain similar covariates for these successive decisions to facilitate comparison, and we avoid regressors that would be endogenous, such as household composition. Other socioeconomic motivations, influences and perceptions could be relevant, as well as distinguishing sociological groups. However, hints about family influence may partly reflect them.

The data used are gathered from the ONFP’s survey PAP-FAM 2001. The main statistical characteristics are summarized in Table 1. One important independent variable, common in the fertility literature, is the *number of desired children* [25–27]*.* In our data, this is recorded through a retrospective question to the women (‘If you think back in time before having your children, how many children would you have liked to have had?’). *A woman’s age* is included as a mere control, not only because it determines its fertility potential, but also because it is associated with diverse lifecycle factors. In this respect, the *husband’s age* is also included. A dummy variable for *urban* areas is incorporated, as fertility often differs substantially between cities and countryside. Moreover, prices are known to vary with urbanization, which affects the cost of raising children. The other covariates can be categorized into measures of educational and career attainment and characteristics of family and social interactions.

**Table 1:**
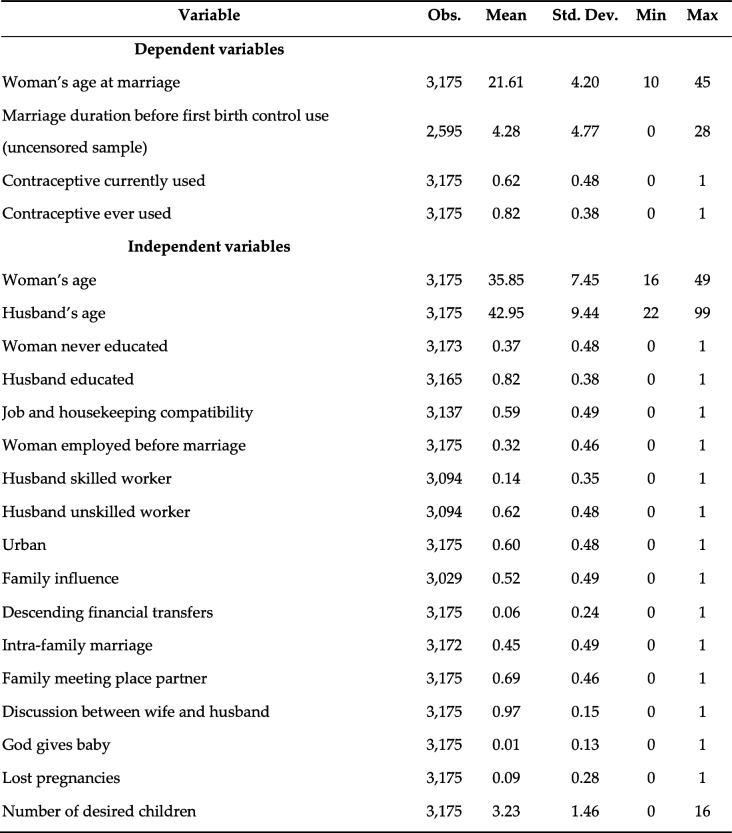
Descriptive statistics

The reference category for ‘Husband skilled worker’ and ‘‘Husband unskilled worker’ is ‘Non-working husband’. The variable ‘Descending financial transfers’ corresponds to couples that receive transfers from both parental couples

Fertility theory, starting with the seminal works by Becker [28–30] has taken into consideration education and job variables that relate to the human capital and opportunity cost of women’s time as determinants of fertility. However, we do not include attainment levels since their effects are not statistically significant, perhaps because these variables are not accurately measured. We only account for schooling through two dummy variables (*woman never educated* and *husband educated*). Furthermore, due missing information on women’s professional status, we employ a binary variable (*woman employed before marriage),* which attenuates the potential endogeneity of female employment in fertility decisions by anchoring the answer in pre-marriage times. Labor market participation before marriage should have occurred before the fertility decisions were made, as having children is tolerated only among married couples. We consider a woman's belief in her ability of simultaneously coping with worker’s and mother’s tasks by including a dummy variable for *job and housekeeping compatibility*. The husband’s professional status is described by two dummy variables for *husband unskilled worker* and *husband skilled worker*, which aggregate job-specific information.

The few authors who incorporated social and familial characteristics confine their attention to father’s characteristics (education, profession, area), as in Wong [31]. We innovate by including covariates related to the family network: a dummy variable for the couples who first met at their family’s home (*family meeting place partner)*, and a dummy variable for the husband being a close relative of his wife (*intra-family marriage*). These variables depict couples with a traditional orientation characterized by closely knit families. In Tunisia, marrying within the family is widespread (42.3 percent), while 63.5 percent of the respondent women first met their future husband at the family’s home. These strong family ties make family influence in the couple’s regulation decisions more likely, and reinforces interactions among family members in general.

We examine the relationship between family involvement and regulation decisions. However, including a variable for the presence of parental childcare may generate a simultaneity bias, as childcare may be spurred by a new birth, which could itself follow some relaxation of birth control. Therefore, we instead use a proxy binary variable that reports whether either a woman’s family or her family-in-law usually intervenes in the nuclear family decisions (*family influence)*. That is, we presume that when the family has the habit of interfering in a couple’s life, this was already the case before any birth. Mahfoudh-Draoui ([32], p. 139) reports that only 8 percent (respectively, 4.5) of households in rural (urban) areas employ external child cares for children under 6 years of age, whereas most childcare is performed by relatives. Likewise, we include the parents’ financial support through a dummy variable (*descending financial transfers*) indicating whether the couple benefits from financial assistance from their parents: 30 (18) percent of the couples regularly receive financial assistance from the husband’s (the wife’s) father or mother; and 6 percent from both parental couples.

In addition, we construct a dummy variable (*discussion between wife and husband*) indicating whether the woman regularly speaks with her husband about his job, financial difficulties, housekeeping problems, or social issues. This variable proxies the degree of understanding between spouses. We also include a dummy variable identifying women who state that children come from God (*God gives baby*), and may shun contraception owing to this belief. Finally, we include a dummy variable for the occurrence of lost pregnancies five years before the survey (*lost pregnancies*) as a proxy for women suffering from a poor reproductive health, a potential reason to limit sexual intercourse.

## Results discussion

We first discuss the econometric approach, and then the estimation results (shown in Table 2 for all regressions).

**Table 2:** Estimation Results

	**Woman’s Age at Marriage**	**Delay till the First Birth Control**	**Contraceptive use**	
			**Probit estimates (marginal effects)**	
	**Weibull duration model**	**Weibull duration model**	**Current contraceptive use**	**Contraceptive ever used**
Woman’s age	-0.0387*** (0.00396)	0.0134*** (0.003)	0.0103*** (0.003)	0.0277*** (0.004)
Husband’s age		-0.00539* (0.002)	0.0011 (0.002)	-0.0039 (0.003)
Woman never educated	0.0178 (0.0751)	-0.231*** (0.058)	-0.150*** (0.05)	-0.222*** (0.06)
Husband educated		0.0475 (0.072)	0.014 (0.06)	0.059 (0.07)
Woman employed before marriage	-0.415*** (0.0617)	0.0736 (0.060)	0.0072 (0.05)	0.009 (0.06)
Job and housekeeping compatibility	-0.166*** (0.0565)	-0.14*** (0.054)	-0.080 (0.04)	-0.147** (0.05)
Husband skilled worker		0.085 (0.093)	-0.019 (0.08)	0.146 (0.10)
Husband unskilled worker		-0.046 (0.063)	-0.029 (0.05)	-0.007 (0.06)
Urban	-0.0700 (0.0686)	0.109** (0.054)	-0.0006 (0.05)	0.069 (0.06)
Intra-family marriage	0.143** (0.0620)	-0.0925* (0.052)	-0.106** (0.05)	-0.080 (0.05)
Family meeting place partner	0.282*** (0.0635)			
Descending financial transfers		-0.309*** (0.106)	-0.352*** (0.09)	-0.290** (0.11)
Family influence		-0.112** (0.052)	-0.146*** (0.04)	-0.158*** (0.05)
Discussion between wife and husband		0.422*** (0.148)	0.155 (0.15)	0.391** (0.16)
God gives baby			-0.583*** (0.18)	-0.475** (0.18)
Lost pregnancies			-0.289*** (0.08)	-0.330*** (0.08)
Number of desired children	0.118*** (0.0167)	-0.0098 (0.016)	-0.0385** (0.01)	-0.025 (0.01)
Constant	-15.919*** (0.398)	-2.187*** (0.266)	0.187 (0.25)	0.102 (0.29)
Weibull parameter (p)	5.36 (0.106)	0.664 (0.0603)		
Observations	3,132	2,901	2,901	2,901

Robust standard errors in parentheses: *** *p* < 0.01, ** *p* < 0.05, * *p* < 0.1

### Age at marriage

The regressions for the *woman’s age at marriage* are specified as Weibull duration model. This model is parsimonious for fitting explanations of duration variables, and allows for increasing and decreasing hazard functions. Its conditioning is based upon the abovementioned covariates. Any obviously endogenous independent variable is omitted. However, since the estimates are based on a cross-section, and finding instruments for all possible regressors suspected to suffer from endogeneity is not feasible, the results should be interpreted as suggestive correlations rather than robust and undeniable causal effects.

Ideally, the population of interest should be that of married women of legal age. Besides, an additional reason to separate married and unmarried women in the analysis is that the marriage event involves such a change in life context that it may generate truly structural changes in behavior. Since only married women are observed in the data, the sample for the marriage age equation is truncated. Indeed, some women were not surveyed because they were not married at the time of the survey, while they may have been or will probably get married later. To explore this issue, OLS and truncated regressions of the marriage’s age are also estimated, in levels and in logarithms, with a few variables that pertain to post-marriage information omitted. Their results show (in the Additional file 1) that the sample truncation that is associated with marriage age is as a matter of fact insignificant for our purpose.

This is the conversion of the Weibull duration model in terms of an accelerated failure time (AFT) model that allows us an easy interpretation of the effects of correlates in terms of changes in failure time. Indeed, the AFT equation, is for individual i: ln(t_i_) = b_0_ + x_i_’b + e_i_, where t_i_ is the failure time, x_i_ is a vector of correlates, b is the vector of coefficients of correlates in the AFT model, b_0_ is an intercept and e_i_ is an error term. Assessing the effect of a change in x_i_ is therefore akin to calculating predictions in a linear model. Thus, a change ∆x_i_ = 1 for a unique correlate, to simplify the discussion, implies a change in failure time equal to ∆t_i_, such that (t_i_ + ∆t_i_)/t_i_ = exp(b). That is: this is the factor by which time-to-failure must be multiplies to obtain a prediction from an initial value, for example the mean of t_i_ for a given subpopulation. Moreover, we have b = -d/p, where d is the vector of coefficient in the Weibull proportional hazard model.^1^

Therefore, the values of the estimated coefficients in the Weibull duration model can be converted into predictions of the effects of changes in the failure times, as we did in the comments. In the estimated models, the estimates of parameter p are 5.36 for the woman’s marriage age model, and 0.663 for the marriage duration till the first birth control.

On the whole, the conjecture that *woman’s age at marriage* serves to delay childbearing is corroborated by these estimates that confirm the substantial influence of typical determinants of fertility demand. Examining distinct age classes would have been interesting, but our sample is too small to do this. Of course, the population’s ideas about marriage may have changed over time, which may imply that the link between *woman’s age at marriage* and birth control is less simple than it may appear. However, the estimated duration model provides us with a simple interpretation grid in which a few control variables, such as age and education, attenuate these concerns.

Interestingly, the *number of desired children* is found to have a significant positive impact on the hazard of marriage, accelerating the contracting of marriage on average. Childbearing is a motivation for the marriage decision.

The women who believe being able to fulfill both job and housekeeping tasks marry significantly later, by almost eight months. Diverse interpretations are possible. Overworked women on their job and at home may be less inclined to marry early, and, thereby to accumulate additional childcare burden. The results also show that, as in Wong [31], (prior to marriage) employed women postpone marriage more than the unemployed women. This is consistent with raising children being a hindrance to career development and even to securing a job. In addition, because of the correlation of a woman’s labor force participation and the minimum husband’s quality that she would accept, her perceived opportunities of marriageable men may be less numerous, which may delay marriage [33, 34].

In contrast to its effect on other decisions, which we discuss below, no significant effect of women’s education on age at marriage is found. However, the few women with higher education in the sample tend to have married later. So, we do not report this variable in the table for consistency with the sets of covariates in the other equations and to avoid drawing conclusions based on too small a subsample.

Last but not least, we find that strong family networks, in particular proxied by the variable ‘*family meeting place of partner*’, increase the hazard of the marriage event. Indeed, marriages tightly controlled by extended families typically take place earlier. Women belonging to such a traditional family that arranges the marriage through meetings spend on average fourteen months fewer on partner search. Since traditional Muslims often marry first cousins or other kin whom they already know, this saves on partners’ time to learn to know each other. Furthermore, ‘*intra-family marriage*’ speeds up marriage occurrence by seven months. Endogamic marriages usually occur much earlier than exogamic marriages. Even though marriages and wedding timings are no longer exclusively arranged by the traditional extended families, these relatives often remain instrumental for these decisions. We now turn to the second-stage decision, which is the *marriage duration till the first birth control*.

### Marriage duration till the first birth control

The regression for the delay before the first birth control is also specified as a Weibull duration model. This is akin to the modelling in Klasen and Launov [35, 36] of the timing of the first birth in the Czech republic. This equation and the equations for the contraceptive uses make sense only for married women since they are the ones possibly using contraception, bar exceptions, in Tunisia. The sample for the delay till the first birth control is right-censored due to the 18 percent of married women who were not observed to have made any birth control attempt at the time of the survey. However, this can be dealt with by specifying the right-censoring in the likelihood function of the Weibull model. All this clarifies the difference between the number of observations in the descriptive statistics and the estimation results. The sample size in these estimations is 3132 for the marriage age equation and 2901 observations for the other equations due to missing values. The estimates of the Weibull model for the delay till the first birth control (*Marriage duration before first birth control use*), measured in years, are shown in Table 2.

The variable *number of desired children* is found not to significantly affect the hazard of the first control. A woman, and her partner, may not start using birth control immediately because they want at least one child, while their final number of children may not necessarily matter for the timing of the first use of contraception since conceptions may be spread over time. Moreover, the couple may decide about a definite number of children only after having had their first child and experienced parenthood. Finally, measures of ideal family size may provide inaccurate information on past motives for having children, as they reflect retrospective opinions that can change. For example, a woman may adjust her fertility target to changes in socioeconomic conditions that alter her perceived costs and benefits of children. In the case of undesired births, ex-post revision of family size preference may also occur through ex-post rationalization. *Husband’s age* weakly negatively affects the delay the first birth control.

Consistently with the previously obtained results for *woman’s age at marriage*, a woman who can accommodate both professional and housekeeping tasks is significantly less inclined to use birth control early, with an almost two-year delay. However, her career plans, as gauged by whether she was *employed before marriage*, do not significantly affect the hazard rate of her initial birth control, perhaps because it is a pre-marriage variable. Indeed, while 37.7 percent of respondents worked before marriage, only 14.4 percent were still working at the time of the survey. Her husband’s professional skill level and education do not significantly affect the delay in the first birth control. This reflects the smaller role that the husband plays in the fertility timing decision and in childcare. In contrast, women’s education matters a great deal. Educated women first use contraceptives earlier after marriage, which contrasts with findings obtained by Bloom and Trussel [36, 37] in the US. Never-educated women delay birth control by as much as 21 months on average. For urban women, contraception occurs on average eight months earlier than the average married woman. Residing in an urban area is associated to lower demand for children and earlier contraception.

Frequent discussions between spouses are significantly and positively associated with a reduction of the span without birth control—by more than five years on average. In couples that communicate a lot, the woman may not feel constrained to have children immediately after marriage to increase her likelihood of retaining her husband. This is the case for couples in which the spouses are enrolled in higher education and decide to complete their studies before having children.

Belonging to a traditional family in which the marriage is arranged by the parents (Intra-family marriage), are found to delay the use of contraceptives by eight months, presumably because conservative values favor a large family size. This is consistent with findings in the literature that family-arranged marriages are associated with higher fertility [37–40]. Moreover, financial parental assistance is found to be correlated with a significant delay in the first birth control, by almost four years. Beyond direct family pressure, parental financial assistance may be correlated to low income that induces a negative income effect, which incite the couple to adjust their family size upward and delay birth control. However, as for the marriage age equation, it is found here that when there is potent influence of the extended family on the couple procreation, it has the effect of encouraging fertility. In a somewhat anthropological approach, Diamond-Smith et al. [35, 40] claim in Nepal that most newly married couples want to delay their first birth, but they feel pressured by in-law and society to have an early child. As a matter of fact, a large progeny, especially with boys, is a conventional and historical objective of traditional parents and grandparents. Arranging meetings and financial supports are some of their means to achieve this goal.

### Contraceptive use

All over the world, the contraceptive prevalence rate is measured for women 15–49 who are married or in union, as well as unmarried women. The regressions for currently using, or having ever used, contraception are specified as probit models.

The last regulation decision considered is captured by two dummy variables indicating whether a contraceptive is currently used (during the survey referring to the question ‘*Are you using any contraceptive method at the moment?’*) or has ever been used (referring to the question ‘*Do you have any experience of contraceptive practice*?’). The estimated marginal effects from the respective probit models are shown in Table 2. We discuss them jointly because the significant effects are often similar. Again, the woman’s age is a mere control.

Economic conjuncture effects may contribute to reducing the use of contraception at some periods, if, as often believed, children’s demand is stimulated by low incomes. Indeed, in that case, age effects for young fertile women may be confounded with year effects since these two variables are directly related in the survey. Economic growth was 8.8 percent on average in the sixties, and 4.2 in the seventies, then it went down to 3.8 during the 1983–96 crisis, to augment again, while only to 5.7 over 1987–2001.

The estimates again highlight familial influences as significant factors. When family members are tightly knit through intra-family marriage, encroachment on marital life, or intergenerational financial transfers, contraceptive devices are less often used, during the survey or before. As in Ghimire and Axinn [38]’s findings in Nepal, the widespread erosion of family-arranged marriages may have weakened resistance to contraception in Tunisia.

Family influence, generally accompanied by opportunities for childcare by grandparents, often reduces the time that a woman has to devote to her children, hence reduces her opportunity cost of having children, and, as a consequence, diminishes her use of contraceptives. Benefiting from family childcare facilitates the coordination of a woman’s roles as worker and housewife, thereby allowing higher fertility [21, 41, 42].As in Ermisch [43], Del Boca [35] and Frini [39], greater family childcare availability fosters fertility. Family influence is associated with a similar decline in contraception use, during the survey, or ever, by approximately 15 percentage points in both cases. Moreover, the availability of parental financial support diminishes the likelihood of having ever used contraception by 29 percentage points, and of using it during the survey by 35 percentage points. Finally, marriages arranged by families are associated with an 11 percentage points lower probability of using contraception during the survey. Again, the sway of the extended family in the procreation process is found to boost fertility, this time through delaying birth control by the couple.

A traditional sociocultural context appears to be unfavorable to birth control. Indeed, the small proportion (1 percent) of women who believe that having a child is a ‘decision made by God’ is less likely to practice contraception by 50 percentage points. As for the delay in the first birth control, frequent communication between spouses, which presumably includes birth control questions, affects birth control in the past (increasing the probability of use by 40 percentage points), but not during the survey. Similar to findings in Link [44], Sharan and Valente [45], and Massenga et al. [46], better communication between the husband and wife increases contraception use, perhaps because they often pondered it jointly.A woman having more education is associated with a greater use of contraception, whether in the past (22 percentage points higher probability of use, relative to women with no education) or during the survey (15 percentage points higher). The literature has long shown that women’s schooling favors more effective and intensive use of contraceptive methods [27, 46–49]. As before, male education does not influence contraception, in contrast to Cochrane and Guilkey’s [50] findings for Tunisia in 1988.

Compatibility between housewife’s and worker’s tasks negatively impacts past contraceptive use (by -15 percentage points), although it is insignificant for current use. This compatibility may matter mostly early in the lifecycle, when the woman attempted to establish her career. If this is the case, it would correspond mostly to past use of contraceptives and explain the results.

However, this is not supported by the other variable on careers (*woman employed before marriage*), which has no significant impact on either contraceptive use. Moreover, the husband’s skill levels, age and residency in urban areas are not associated with fertility control in this case.

The number of children desired is slightly negatively associated with the probability of current contraceptive use (-4 percentage points per additional child), as in Bollen et al. [51] for 1988 Tunisia, and it does not significantly affect past use. The latter may be because fertility goals may change over time. Finally, lost pregnancies, which may be a sign of health problems, seem to induce women to avoid sexual relationships, and thereby reduce the probability of contraceptive uses by almost one-third, in the short and long run.

## Conclusion

In this investigation, we consider several consecutive birth control decisions made by married women and their families in Tunisia: age at marriage, marriage duration at the first contraceptive use, and past and current contraceptive use.

Although perfect causal inference is not possible with the cross-sectional data that is used, the correlations obtained suggest explanations that call for additional collection efforts to better observe the lifecycle decisions of family members and the interactions with the extended family. Beyond, the effects of diverse covariates, our main finding is the ubiquitous influence of the spouses’ extended families on a woman’s birth control, as measured with the above decisions. They appear as relevant determinants of fertility choices mostly through their arranging of, often endogamous, marriages, and the financial transfers to their married children.

On the one hand, women belonging to traditional families that arrange the marriage through home meetings spend much less time on partner search. Moreover, they delay the first use of contraceptives after marriage by eight months, and they have a lower probability of using contraception. Endogamic marriages typically occur much earlier than exogamic marriages. When family members are tightly knit through endogamic marriage and encroachment on marital life, contraceptive devices are less often used. When either a woman’s family or her family-in-law are involved in the nuclear family decisions, one observes, on average, less contraceptive use. On the other hand, financial parental assistance is correlated with a significant delay in the first birth control, and a rarer use of contraceptive devices.

Over time, the erosion of the influence of extended family is linked to the rise of individualism, especially in urban areas or among the educated classes. This evolution is accelerated by the use of new information communication technologies, from internet social networks to mobile phones, that makes Muslim youth in Tunisia increasingly similar to their non-Muslim generational counterparts elsewhere in the world. The growing desire of young generations of freedom from the traditional authority of elders and of the extended family leads them to more autonomy in the decision-making process about procreation. However, reactionary forces are also at play that promote the influence of the extended family: the economic crises that increase the need for family support, the new communication tools with the elderlies through social networks, the rising traditional and radical religious movements. What was found is that the extended family is still influential in the procreation process in Tunisia.

Even though we cannot assess—using these data—the direct effects of public contraception services, it is nevertheless valuable to draw tentative policy lessons. The results show that policy-makers should take greater consideration of the extended family when designing family planning programs. For example, surveys to monitor fertility could be directed not only toward women but also to husbands and to extended families. Moreover, media and advertising campaigns could also be targeted at men and families, not just women, even though these campaigns also contribute to diminishing the influence of extended families. This would assist couples in dealing with conflicting fertility norms and objectives within their extended families. They may also make these relatives accept to grant more freedom to the women in their own procreation choice. A similar program targeting male partners has already been put in place in Tunisia [52].

The observations that we have proposed in this work about the fertility and birth control in Tunisia, characterized both by generational changes and the persisting influence of the extended families, are likely to be generalizable to other contexts, in MENA countries and elsewhere. This influence is not disappearing, but merely reduced. New ideologies, new technologies and solidarity needs in difficult economic times may contribute to revive it. As a consequence, the role of extended families could be taken more seriously in family planning programs almost everywhere.

Finally, another lesson from these reflections is that more studies on the interactions of generations in couples’ fertility processes would be desirable, both from theoretical and empirical points of views. Currently, this remains a highly under-researched area.

## Supplementary Information

**Additional file 1.**

## Data Availability

Publicly available datasets were analyzed in this study. These general data can be found here: Office National du Planning Familial, Rue 7051, Tunis, Tunisia. The datasets used and analyzed during the current study are available using the direct accessible link: https://www.dropbox.com/scl/fo/e5ahi6s6iprn8i7u7v59j/h?dl=0&rlkey=yvx3zoofexv6gmcl8xsz49tge.

## Abbreviations

AFT: Accelerated failure time
cdf: Cumulative density function
INS: Institut National des Statistiques
PAP-FAM: Enquête nationale sur la santé familiale et les mutations sociales
ONFP: Office National de la Famille et de la Population
QED: Quid es demonstratum

## Footnotes

1 Proof: On the one hand, in the Weibull model, the AFT specification corresponds to e_i_ = ln(k_i_), where k_i_ follows a Weibull(b_0_,p) distribution that has a cdf F(k) = 1 – exp(-[{exp(-b_0_})k]^p^), and p is a real-valued parameter. Then, E(ln(t_i_) | x_i_) = b_0_ + x_i_’b + Г’(1)/p, where Г(.) is the Gamma function, and the OLS estimates of b are inbiased under usual conditions that boil down to the exogeneity of the correlates. The survival function can be expressed, from the cdf, as: S(t_i_ | x_i_) = exp(-[{exp(-b_0_—x_i_’b})t_i_]^p^). On the other hand, the baseline hazard in the Weibull proportional hazard model is. h_0_(t) = pt^p−1^exp(d_0_), which yields a hazard function h(t) = pt^p−1^exp(d_0_ + x_i_’d), where (the same) p, d_0_ and d are parameters. By integration and transformation, one obtains the survival function. S(t_i_ | x_i_) = exp(-[{exp(d_0_ + x_i_’d})t_i_]^p^). Therefore, by comparing the two expressions of the survival function, we have: b = -d/p. QED.
