# Supplementary material for: Revisiting fertility regulation and family ties in Tunisia
Source: BMC Pregnancy Childbirth. 2023 Feb 1;23:88. doi: 10.1186/s12884-023-05408-9 (PMC9890736; doi:10.1186/s12884-023-05408-9)
Supplement: Supplementary file 1 — Additional file 1. [file 12884_2023_5408_MOESM1_ESM.docx]

**Appendix : Evidence of insignificant truncation**

| **Woman’s Age at Marriage** | | | | | | |
| --- | --- | --- | --- | --- | --- | --- |
|  | OLS  (a) | Truncated regression  (b) | | OLS in Logs  (c) | Truncated regression in Logs  (d) | Weibull duration  Model (e) |
| Woman’s age  (or Log age) | .0571***  (.0100) | -.0325***  (.0123) | .0861***  (.0149) | | -.0733***  (.0199) | -0.0388***  (0.00397) |
| Woman never educated | -.355**  (.166) | -.377**  (.180) | -.0209***  (.00732) | | -.0206***  (.00813) | 0.0178  (0.0751) |
| Job and housekeeping compatibility | .225  (.146) | .249  (.159) | .00832  (.00642) | | .00870  (.00721) | -0.166***  (0.0565) |
| Woman employed before marriage | 1.77***  (.160) | 1.83***  (.178) | .0813***  (.00703) | | .0870***  (.00809) | -0.415***  (0.0617) |
| Urban | .275*  (.161) | .269  (.177) | .0126*  (.00708) | | .0143*  (.00801) | -0.0701  (0.0686) |
| Family meeting place partner | -.796***  (.149) | -.917***  (.163) | -.0454***  (.00678) | | -.0407***  (.00737) | 0.143**  (0.0620) |
| Intra-family marriage | -1.01***  (.154) | -1.12***  (.170) | -.0348***  (.00655) | | -.0519***  (.00771) | 0.282***  (0.0635) |
| Number of desired children ​ | -.284***  (.0501) | -.289***  (.0537) | -.0122***  (.00220) | | -.0123***  (.00241) | 0.118***  (0.0167) |
| Constant | 20.8***  (.420) | 24.5***  (.521) | 2.80***  (.0529) | | 3.39***  (.0718) | -15.9***  (0.398) |
|  |  |  |  | |  |  |
| Weibull parameter log(p) |  |  |  | |  | 1.67  (0.0198) |
|  |  |  |  | |  |  |
| Observations | 3,132 | 3,132 | 3,132 | | 3,132 | 3,132 |

Robust standard errors in parentheses: *** p<0.01, ** p<0.05, * p<0.

For showing that the truncation is insignificant in that case, we estimate accelerated failure time (AFT) models that can be defined by the equation: log(t_i_) = x_i_^’^b + f_i_, where t_i_ is age at marriage, x_i_ are explanatory factors, b is a vector of parameter to estimate, and f_i_ is an error term, for observations i=1,…,n. An AFT model provides an alternative to proportional hazard models that assume that a covariate changes multiplicatively the hazard by some constant. Instead, in an AFT model covariates accelerates the life span by some constant. Because they can be estimated in linear regressions, AFT models are robust to exogenous omitted covariates, and do not depend asymptotically on the choice of the parametric distribution of errors. Finally, the Weibull model can be reformulated as an AFT model. However, since AFT models in general do not imply constant hazard ratio, a popular assumption in social sciences, they are viewed here rather as an investigation device rather than an alternative to proportional hazard models. If there is no truncation, this equation can be estimated consistently by using OLS, for any reasonable distributions of errors, assuming that all factors are exogenous. To allow for the truncation, a truncated regression is estimated using the maximum likelihood method, by assuming that the f_i_ follows a normal distribution. If f_i_ is normal, then the AFT model is a lognormal model. By changing the functional form of the dependent variable, the restriction on the error distribution in the t_i_ model can be varied. For example, an equation with t_i_ in levels is also estimated, using both OLS and truncated regressions: t_i_ = x_i_^’^b + f_i_. Across all the tried estimation results (not all shown), there is a general correspondence in the signs and significance of the estimated coefficients, except for the age coefficient, for all estimation methods, including for the Weibull duration model that is related to the opposite of t_i_. Indeed, increasing the hazard function of marriage corresponds to decreasing the age of marriage.

Therefore, a qualitative agreement is found for all estimation results for the marriage’s age determinants, whether or not truncation is taken into account. Moreover, examining the precise estimation results in the table, for column (a) versus column (b), and column (c) versus column (d), shows that the effect of the truncation is likely to be insignificant in these data, for any included factor, except for age for which the estimated coefficient changes sign after correction for truncation. The latter is not surprising because most women yet-to-be-married are young, and the truncation is correlated with age. Therefore, in our comments we focus on the Weibull duration model estimates, in column (e), without truncation correction. However, when moving from AFT models to the Weibull model, the estimated coefficient of the variable ‘Woman never educated’ becomes insignificant, whereas the effect of ‘Job and housekeeping compatibility’ becomes very significant. This may uphold the use of the Weibull model instead of mere regressions.
